# A Stable Metal Chalcogenide Cluster-Based Framework Decorated with Transition Metal Complexes for an Efficient Electrocatalytic O_2_ Reduction Reaction

**DOI:** 10.3390/nano15151186

**Published:** 2025-08-01

**Authors:** Xiang Wang, Juan Li, Tao Wu

**Affiliations:** 1 College of New Energy Materials and Chemistry, Leshan Normal University, Leshan 614000, China; 2 Leshan West Silicon Materials Photovoltaic and New Energy Industry Technology Research Institute, Leshan 614000, China; 3 College of Chemistry, Chemical Engineering and Materials Science, Soochow University, Suzhou 215123, China; 4 Hongguang Middle School, Nanjing 210018, China; 5 Guangdong Provincial Key Laboratory of Functional Supramolecular Coordination Materials and Applications, College of Chemistry and Materials Science, Jinan University, Guangzhou 510632, China

**Keywords:** metal chalcogenide cluster-based framework, oxygen reduction reaction, non-Pt electrocatalysts manganese, transition metal complexes

## Abstract

Highly efficient and stable non-Pt-based electrocatalysts for oxygen reduction reactions (ORRs) are highly desirable in energy conversion and storage systems. Herein, we report a hydrothermally synthesized metal chalcogenide cluster-based framework (NCF-3-Mn), which is decorated with transition metal complexes ([Mn(TEPA)]^2+^, TEPA = tetraethylenepentamine), for an electrocatalytic O_2_ reduction reaction (ORR). Benefitting from the abundant Mn-S bonds and Mn-N-C structures in NCF-3-Mn, it was found that NCF-3-Mn displayed a high onset potential (0.90 V) and an efficient four-electron transfer reaction pathway, which are much better than those of its analogue framework (T2-GaSbS). Moreover, NCF-3-Mn also exhibited a considerable long-term stability and methanol resistance toward ORRs. This work will present new opportunities for exploring the utilization of chalcogenide frameworks as novel non-Pt electrocatalysts for ORRs.

## 1. Introduction

With ever-increasing environmental pollution and energy scarcity, great efforts have been devoted to various sustainable energy conversion and storage systems, such as fuel cells, metal–air batteries, water-splitting devices, etc. [1,2,3,4,5,6,7,8,9,10]. However, as the key semi-reaction in these devices, the oxygen reduction reaction (ORR) is up against inherent sluggish kinetics, which leads to high overpotential and poor efficiency of related devices [1,6,9].

To solve this problem, Pt and its derivatives are considered to be the state-of-the-art electrocatalysts toward ORRs so far. Nevertheless, suffering from exorbitant prices, scarce resources and deactivation or alterations in structure over extended periods, the market feasibility of these catalysts is limited. Consequently, designing and synthesizing cost-effective and earth-abundant non-precious metal-based electrocatalysts with excellent catalytic activity and stability is crucially important [2,3,11,12].

Toward this target, numerous non-precious metal catalysts with low cost and good electrocatalytic performance have been extensively developed over the last few decades [1,2,3,11,12,13]. Among these non-precious metal catalysts, transition metal–nitrogen–carbon (M-N-C)-based or -contained catalysts [14,15,16,17,18,19,20,21], especially Fe-N-C catalysts [22,23,24], have been widely investigated and considered as the most promising precious metal-free catalysts, for their atomically dispersed M–N_x_–C_y_ active sites can readily activate O_2_ and subsequently break the O–O bond with a lower energy barrier and thereby facilitate the ORR [14,15,16,18].

Despite extensive research on these catalysts, the local coordination environments of the M-N-C catalysts are difficult to control because of the various surface sites, such as structural defects generated during the preparation process for heterogeneous catalysts [18,23,25], which usually make it difficult to establish a precise structure–activity relationship and even make the actual active sites controversial [25]. Therefore, exploring novel efficient non-precious metal catalysts with definite structure and precise catalytic sites for the ORR is of great significance [14,15].

Metal chalcogenide cluster-based frameworks (MCCFs), as the hollowed-out counterparts of dense-phase II–VI semiconductors, are assembled by interlinked negatively charged metal chalcogenide clusters and compensated by different cations [26,27]. Due to their highly ordered porosity, intrinsic semiconductivity, multifarious coordination modes, different cluster types and tunable elemental composition, in recent decades, MCCFs have shown promising application prospects in various fields, such as ion exchange, gas sorption, photoluminescence and photocatalysis, etc. [26,27,28].

When used as catalysts, MCCFs could be ideal models for exploring the structure–activity relationship and active sites as a result of their well-defined structure [26,27]. Moreover, the negatively charged metal chalcogenide cluster in the framework of this material could provide a container for electron transfer in electrocatalytic processes, as well as adsorbing more oxygen molecules, which is beneficial for the electrocatalytic ORR [26,27]. Consequently, the ORR activities of several MCCFs have been explored in recent years. For instance, Wu’s group has reported a series of 3D MCCFs for ORRs [26,27], including CSZ-5-InSe, SOF-27, SOF-25/28, NCF-4, MCOF-1/2, SOF-20/21 and Mn_5_Sb_6_S_15_(N_2_H_4_)_6_. Additionally, Sun et al. recently studied the electrocatalytic ORR activity of two antimony-based and three non-supertetrahedral metal chalcogenide cluster compounds with low-dimensional structures [29,30].

Notably, only NCF-4 displayed promising catalytic properties for ORRs whereas the other aforementioned MCCFs are unsatisfactory in terms of onset potential (*E*_onset_), half-wave potential (*E*_1/2_), electron transfer number (*n*) and diffusion-limited current density (*j*_L_) [31]. It is well-known that manganese-based electrocatalysts usually exhibited high ORR activity [14,15,16,17,32] and thus the better ORR performance of NCF-4 is believed to originate from the M-N-C analogues formed by integrated Mn complexes and the incorporation of Mn elements into the clusters [14,15,16], whereas other MCCFs merely comprise clusters composed of In/Sn/Sb elements and the electrocatalytic ORR activity of these elements is typically low [29,30].

However, as the coexisting Mn complexes and Mn sites located on the surface of clusters in NCF-4 may both contribute to the good catalytic performance of ORRs, it makes the catalytic active sites in NCF-4 ambiguous [31], which impedes the electrocatalytic applications of this material. Hence, to develop the electrocatalytic ORR applications of MCCFs, it is necessary to seek an MCCF with distinct catalytic active sites and high activity.

Herein, an MCCF decorated with transition metal complexes (NCF-3-Mn) was synthesized via a solvothermal method and explored for ORRs while another MCCF called T2-GaSbS, with a similar structure yet without transition metal complexes, was chosen as a control sample for comparison. Details of the differences in structure and composition between NCF-3-Mn and T2-GaSbS are discussed in the following Results and Discussion. Owing to the introduction of transition metal complexes [Mn(TEPA)]^2+^, there are many Mn-S bonds and Mn-N-C structures in the framework of this material, which is favorable for electrocatalytic ORRs [14,15,16,18]. As a result, NCF-3-Mn displayed excellent electrocatalytic ORR performance in terms of *E*_onset_ up to 0.90 V (vs. RHE), an electron transfer number of nearly four (3.95), as well as robust stability and good methanol tolerance in alkaline solution, showing the promising application of MCCFs in ORR-related fields.

## 2. Materials and Methods

The synthesis of NCF-3-Mn and T2-GaSbS was prepared according to the method in the corresponding literature [33,34].

The surface morphology and elemental composition were performed on a scanning electron microscope (SEM) equipped with an energy dispersive spectroscopy (EDS) detector under an accelerating voltage of 25 kV and 40 s accumulation time. The structural characteristics and phase purity were analyzed through powder X-ray diffraction by a desktop diffractometer (D2 PHASER, Bruker, Berlin, Germany) using Cu-Kα (λ = 1.54184 Å) radiation operated at 30 kV and 10 mA. X-ray photoelectron spectroscopy (XPS) was used to analyze the chemical states of surface elements and collected on a Leeman prodigy spectrometer equipped with a monochromatic Al Kα X-ray source and a concentric hemispherical analyzer.

All electrochemical experiments were tested in a stand three-electrode electrochemical system via a computer-controlled electrochemical workstation (CHI-760e) at room temperature (RT). A 4 mm diameter rotating disk electrode (RDE) with a glassy carbon (GC) disk, a no-leak Ag/AgCl electrode (saturated with KCl) and a graphite rod were used as the working, reference and auxiliary electrodes, respectively. The electrolytes were 0.1 M KOH prepared by KOH of guaranteed reagent and ultrapure water (18.2 MΩ/cm). All the potentials were reported versus a reversible hydrogen electrode (RHE) according to the Nernst equation: *E* (V vs. RHE) = *E* (V vs. Ag/AgCl) + 0.197 V + 0.0591 × pH.

To prepare the working electrode, the RDE was mirror-polished with alumina slurry firstly. Then, after the synthesized sample and carbon black (CB, Vulcan XC-72) were mixed and fully ground in an agate mortar, 12 mg of the mixture and 20 μL 5 wt% Nafion solution were suspended in 1 mL H_2_O/EtOH solution (V/V = 1:1) and ultrasonicated for ca. 30 min to obtain a homogenous catalyst ink. Finally, 5 µL of the as-prepared ink was dropped onto the pre-cleaned surface of the RDE (loading amount:0.234 mg·cm^−2^) and dried naturally at RT before further use.

Details for ORRs and other electrochemical measurements are provided in the Supporting Information.

## 3. Results and Discussion

### 3.1. Structure Characterization

**Figure 1:**
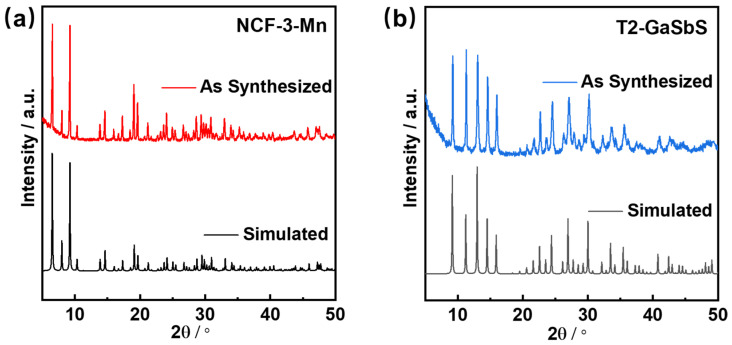
The simulated and experimental XRD patterns of (**a**) NCF-3-Mn and (**b**) T2-GasbS.

In accordance with the literature [33,34], we synthesized NCF-3-Mn and T2-GaSbS solvothermally and XRD was firstly adopted to characterize the phase purity and chemical structure of the two synthesized samples. As shown in Figure 1, the XRD patterns of both samples were consistent with their simulated patterns, without diffraction peaks of impurities, demonstrating the purity of successfully synthesized NCF-3-Mn and T2-GaSbS.

**Figure 2:**
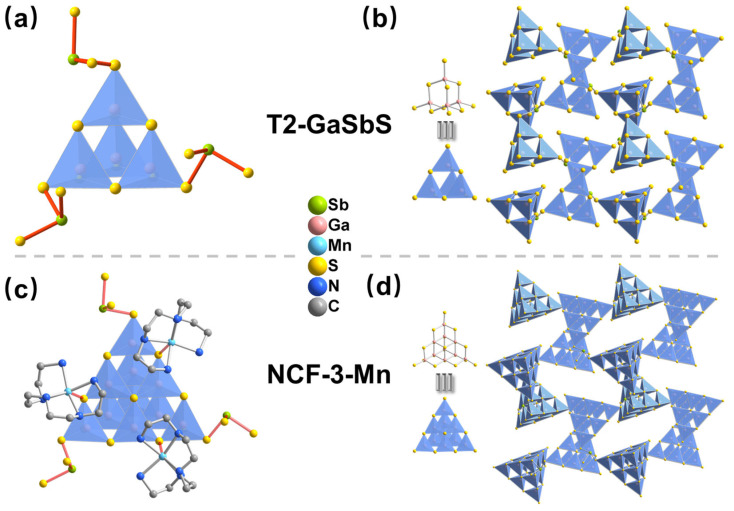
Structure of (**a**) the three-coordinated T2 (*μ*_3_-T2) cluster surrounded three [SbS_3_] linkers, (**b**) 3D open-framework of T2-GaSbS, (**c**) the three-coordinated T3 (*μ*_3_-T3) cluster surrounded with [Mn(TEPA)]^2+^ complexes and three [SbS_3_] linkers and (**d**) 3D open-framework of NCF-3-Mn.

As shown in Figure 2a,c, three vertices S on the same triangular surface in the T2 cluster are connected to a single Sb^3+^ ion, each Sb^3+^ ion is also connected to three T2 clusters, and the topology is the same as that of NCF-3-Mn (Figure 2b,d), except three [Mn(TEPA)]^2+^ complexes linked to three S atoms each at three edges near the corner of each T3 cluster, creating many well-defined Mn-S bonds and a Mn-N-C structure in the framework of NCF-3-Mn. This explicit structural difference makes T2-GaSbS the most ideal comparison model reported so far to explore whether the Mn-S bond and the Mn-N-C structure could greatly improve the catalytic performance since all attempts to date to synthesize new compounds merely comprised supertetrahedral T3 clusters of [Ga_10_SbS_19_SH] connected by single Sb^3+^ ions have failed [33].

### 3.2. Surface Morphology and Chemical State Characterization

**Figure 3:**
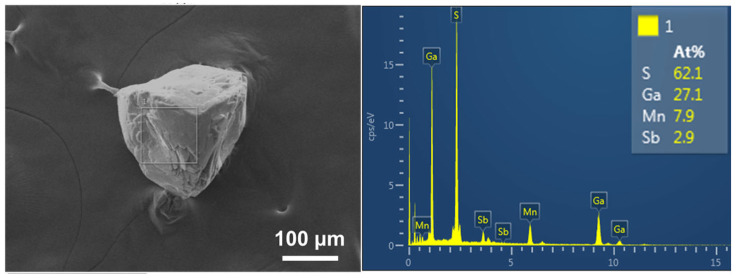
SEM image (**left**) and corresponding EDS spectrum (**right**) of as-synthesized NCF-3.

As shown in Figure 3, NCF-3-Mn shows a smooth surface with a triangular pyramid shape. To investigate the ratio of compositions in NCF-3-Mn, EDS was further performed. The results confirmed the presence of Mn, Ga, Sb and S elements, and the ratio of elements (Ga:Sb:Mn) was about 9.8:1:2.87:21.42, which is in accordance with the theoretical value of 10:1:3:20, further demonstrating the successful synthesis of NCF-3-Mn.

**Figure 4:**
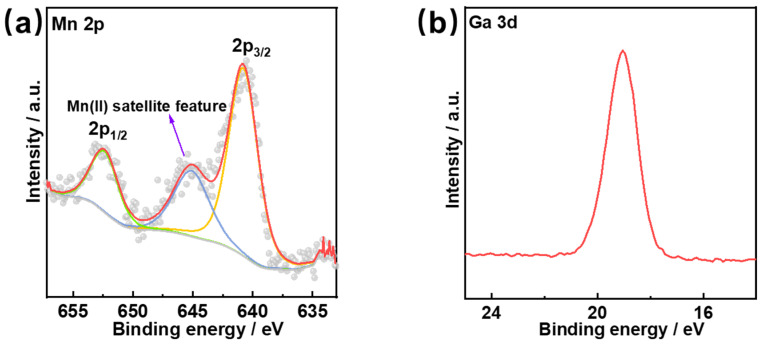

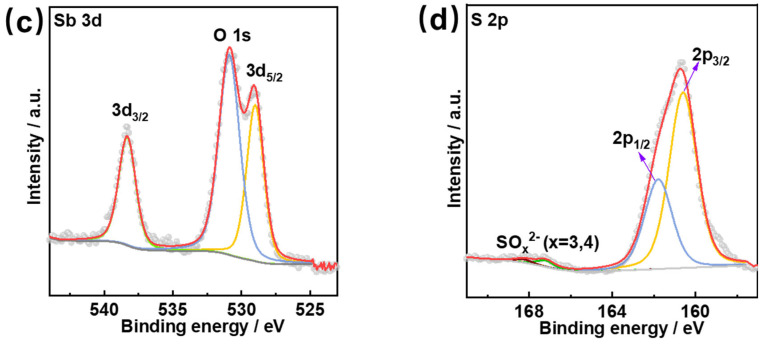
High-resolution XPS spectra of (**a**) Mn 2p, (**b**) Ga 3d, (**c**) Sb 5d and (**d**) S 2p in NCF-3-Mn.

XPS was utilized to analyze the surface chemical composition and states of the valence state of Mn, Ga, Sb and S and surface chemical composition in NCF-3-Mn. The full-scan XPS spectrum of as-synthesized NCF-3-Mn indicates the presence of C, N, Mn, Ga, Sb and S elements (Figure S1), which is consistent with the aforementioned EDS test result. As is well known, the oxidation states of Mn have close relations with the ORR performance [35], and as depicted in the high-resolution XPS spectrum of the Mn 2p region (Figure 4a), two strong peaks at 640.7 eV and 652.5 eV were observed, which are assigned to Mn 2p_3/2_ and Mn 2p_1/2_ of MnS [31,35,36], respectively. Moreover, the peak at 645.2 eV is the typical satellite peak of Mn(II) [31,35,36]. Thus, the valence state of Mn in NCF-3-Mn is +2.0, which is in line with the single crystal data of NCF-3-Mn according to the literature [33]. In Figure 4b, the peak at 19.1 eV is assigned to the Ga-S bond in the NCF-3-Mn. As for the Sb element, the binding energies of 529.1 eV and 538.4 eV in Figure 4c correspond to Sb 3d_5/2_ and Sb 3d_3/2_, respectively, indicating that the valence state of the Sb element is positive trivalent [36]. As shown in Figure 4d, there are four signals in the S 2p spectrum, corresponding to the S 2p_3/2_ and S 2p_1/2_ orbitals of metal sulphides (160.6 eV and 161.8 eV) and a little highly oxidized sulfur species (167.3 eV and 168.3 eV), respectively [35,37]. The highly oxidized states of small amounts of sulfur species may be caused by the oxidation of S exposed in air [35,37].

### 3.3. Electrocatalytic Performance of ORRs

**Figure 5:**
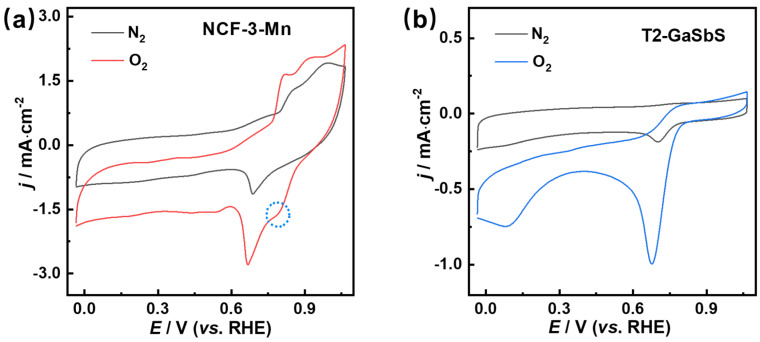
CV curves of (**a**) NCF-3-Mn and (**b**) T2-GaSbS in N_2_/O_2_-saturated 0.1 M KOH.

As MnS is generally considered to be an effective ORR electrocatalyst [31,35], and there are many Mn-S bonds and a Mn-N-C structure existed in NCF-3-Mn, we thought NCF-3-Mn may possess promising potential ORR activity. Hence, the ORR performance of NCF-3-Mn was explored in 0.1 M KOH solution at room temperature and T2-GaSbS and CB were used as the reference samples. Cyclic voltammetry (CV) experiments were firstly conducted to evaluate the ORR activity. As shown in Figure 5a, NCF-3-Mn clearly displayed a distinct enhanced cathodic reduction peak at 0.78 V vs. RHE in O_2_-saturated 0.1 M KOH, while no obvious reduction peak at this position was observed in the CV curve under N_2_-saturated solution, suggesting NCF-3-Mn possesses ORR activity. For comparison, as shown in Figure 5b, T2-GaSbS has a reduction peak at 0.68 V vs. RHE under O_2_-saturated atmosphere, whereas a very weak reduction peak at this position displayed in the N_2_-saturated CV curve, indicating T2-GaSbS may also possess a certain ORR activity. However, the reduction peak potential of NCF-3-Mn is higher than that of T2-GaSbS, and the peak current density of the former is also higher (NCF-3-Mn: −1.66 mA·cm^−2^, T2-GaSbS: −1.00 mA·cm^−2^), clearly manifesting that the ORR activity of NCF-3-Mn is higher than that of T2-GaSbS.

**Figure 6:**
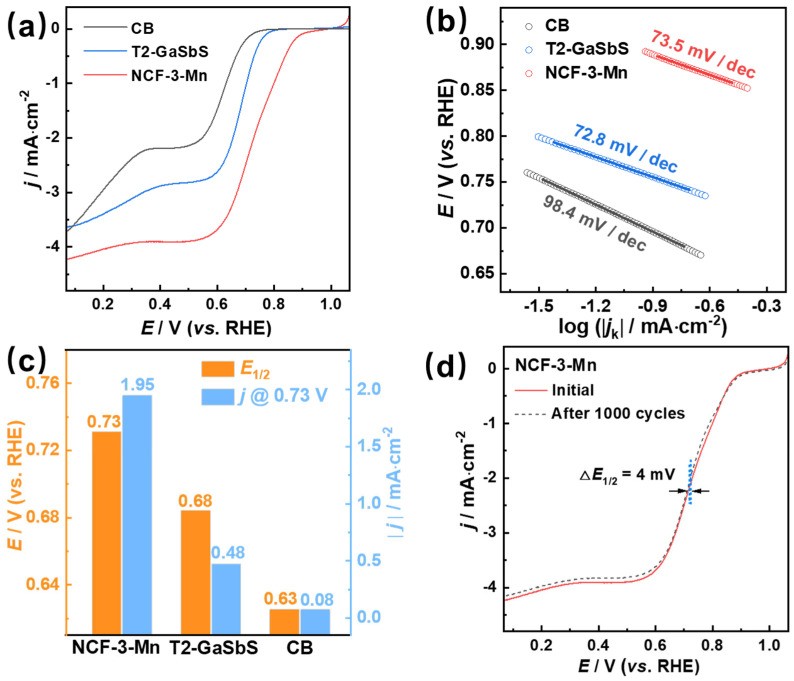
Comparision of (**a**) LSV curves, (**b**) corresponding Tafel slopes, (**c**) current density (at 0.73 V vs. RHE) and *E*_1/2_ of NCF-3-Mn, T2-GaSbS and CB for the ORR at 1600 rpm in O_2_-saturated 0.1 M KOH. (**d**) LSV curves of NCF-3 before and after 1000 CV cycles.

Furthermore, linear sweep voltammetry (LSV) was employed to assess the electrocatalytic activity of NCF-3-Mn and other reference samples through ORR polarization curves. As displayed in Figure 6a, according to the LSV analysis, the onset potential (*E*_onset_) (*j* = −0.1 mA·cm^−2^), half-wave potential (*E*_1/2_) and diffusion-limited current density (*j*_L_) of NCF-3-Mn are 0.90 V, 0.73 V and 3.98 mA·cm^−2^, respectively, which are clearly superior to those of T2-GaSbS (0.78 V, 0.68 V, 2.83 mA·cm^−2^) and CB (0.75 V, 0.63 V, 2.20 mA·cm^−2^). Moreover, the mass activity (*j*_MA_) and kinetic current density (*j*_K_) at 0.73 V of NCF-3-Mn are 8.41 mA/mg and 3.90 mA·cm^−2^, which are comparable to those of commercial Pt/C (10 wt%) (*E*_1/2_ = 0.70 V, *j*_L_ = 3.8 mA·cm^−2^, *j*_MA@0.73 V_ = 8.79 mA/mg, *j*_K@0.73 V_ = 3.74 mA·cm^−2^) according to the reported literature [31,36], demonstrating that the decorating of [Mn(TEPA)]^2+^ complexes on T3 clusters is favorable to improve the ORR catalytic performance. In addition, in contrast to a diffusion-limited region that was observed in the LSV curve of NCF-3-Mn, a double-plateau profile could be observed in the LSV curves of T2-GaSbS and CB, implying the ORR for T2-GaSbS and CB proceeds mainly via a two-electron dominant pathway [38].

In addition, corresponding Tafel slopes of kinetic current at low-overpotential regions for as-synthesized samples were obtained. As shown in Figure 6a, the outstanding ORR performance of NCF-3-Mn was confirmed by the relative low Tafel slope (73.5 mV/dec). Although this value is a little larger than that of T2-GaSbS (72.8 mV/dec), it is much smaller than that of CB (98.4 mV/dec), and smaller than that of commercial 20% Pt/C (82.6 mV/dec) according to the literature [39], manifesting an intrinsic faster kinetics process of NCF-3-Mn towards the ORR at low overpotential. According to the literature [38], the rate-determining step and kinetics of electron transfer involved in the ORR could be reflected by the Tafel slope value, while the Tafel slope of NCF-3-Mn is 73.5 mV/dec, which is close to 60 mV/dec, indicating that the rate-determining step in the ORR is the protonation of superoxide ($O_{2}^{-}$) to form peroxide (${HO}_{2}^{-}$) in the alkaline solution [38]. This fast kinetics might be mainly ascribed to the presence of a Mn-N-C structure and Mn-S bonds that could serve as the exposed active sites for activating O_2_ and subsequently breaking the O–O bond with a lower energy barrier and thereby facilitating the electron transfer involved in the ORR [14,15,16,17,18,22,23,25]. Specifically, as shown in Figure 6c, the current density of NCF-3-Mn at *E*_1/2_ (0.73 V) is 1.95 mA·cm^−2^, which is 4~24 times higher than that of T2-GaSbS and CB, demonstrating NCF-3-Mn is the best ORR electrocatalyst among the three samples.

To further determine the intrinsic activity of the catalysts, the electrochemical surface area (ECSA) during the catalytic process was evaluated from the electric double layer capacitance (C*_dl_*) by scanning over a small potential range of 0.9813–1.0813 V (vs. RHE) under different scan rates (Figure S2) [37,39]. As displayed in Figure S3, the C*_dl_* value of NCF-3-Mn obtained from Figure S2a is 4.84 mF cm^−2^, which is about 5.26 times larger than that of T2-GaSbS (0.92 mF cm^−2^). Since the electrochemical surface area is positively correlated with C*_dl_* [39], NCF-3-Mn has a larger electrochemical surface area; that is, more active sites can be exposed in the ORR, which also corresponds with its better catalytic performance.

Aside from ORR activity, as long-time stability is one of the important indicators to high-performance ORR catalysts, both NCF-3-Mn and T2-GaSbS undergo repeated CV cycles in O_2_-saturated 0.1 M KOH. As can be seen in Figure 6d, the *E*_1/2_ of NCF-3-Mn shifted negatively merely about 4 mV after 1000 CV cycles, while the *E*_1/2_ of T2-GaSbS negatively shifted by ca. 66 mV after the same processing procedure (Figure S4a), demonstrating the better stability of NCF-3-Mn. Meanwhile, the stability of NCF-3-Mn is also evaluated via a long-term chronoamperometric experiment and the current density of NCF-3-Mn decreased less than 25% after 40,000 s under a static potential (0.5 V vs. RHE), which is better than the previously reported commercial 10 wt% Pt/C [31,36], showcasing the comparable electrochemical stability of NCF-3-Mn for prolonged use toward ORRs. Moreover, NCF-3-Mn also displays superb methanol resistance ability. As shown in Figure S5, no remarkable current density drop was observed after adding 2 mL of 3.0 M methanol solution to the electrolyte, which is in stark contrast to the previously reported Pt/C catalyst that displayed a sharp current decrease with the addition of methanol into the electrolyte [14,15,16,17,39]. In summary, stable and durable NCF-3-Mn could be a promising candidate for ORRs under actual conditions.

**Figure 7:**
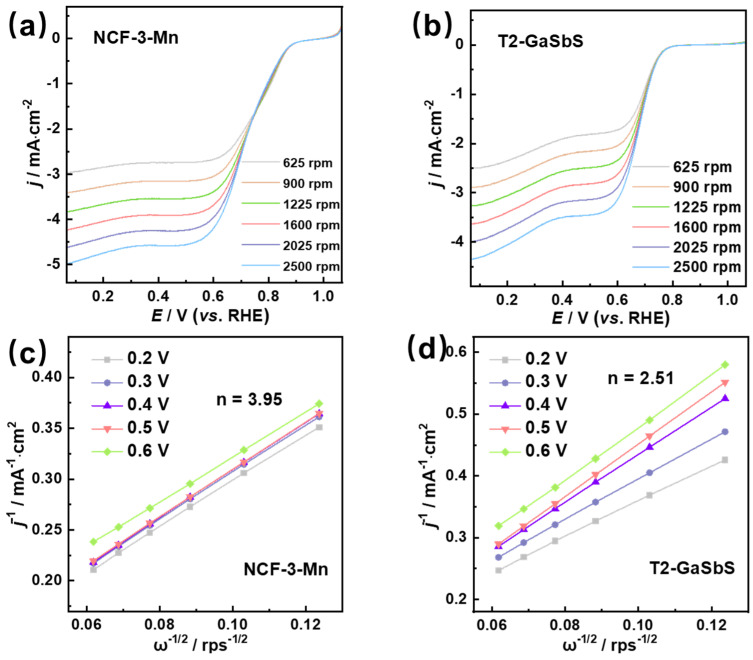
LSV curves of (**a**) NCF-3-Mn and (**b**) T2-GaSbS at the rotation speed from 625 rpm to 2500 rpm with a scan rate of 5 mV·s^−1^ in O_2_-saturated 0.1 M KOH solution. The corresponding K-L (*j*^−1^ vs. ω^−1/2^) plots at different potentials of (**c**) NCF-3-Mn and (**d**) T2-GaSbS.

In addition, the ORR kinetics of the as-synthesized NCF-3-Mn and T2-GaSbS were also investigated through rotating disk electrode (RDE) measurements with different rotating speeds. As presented in Figure 7a,b, both the LSV curves of NCF-3-Mn and T2-GaSbS at different rotational speeds (625–2500 rpm) have a similar change law. The current densities of the two catalysts enhanced as the rotation speed increased, arising from the shortened diffusion distance of O_2_ at a high rotating speed, while the onset potentials were maintained under different rotation speeds. Additionally, the current of T2-GaSbS did not reach the diffusion-limited plateau, while the ORR for NCF-3-Mn was allowed to reach the diffusion-limited region (0.2–0.6 V vs. RHE) from the kinetic-limited region (0.6–0.9 V vs. RHE) due to the faster kinetics [38].

Corresponding Koutechy–Levich (K-L) curves of the two catalysts in the diffusion-limited region were well fitted to evaluate the electron transfer number (Figure 7c,d). As shown in Figure 7c, the slopes of the five K-L curves of NCF-3-Mn coincide, indicating that its ORR process follows first-order reaction kinetics toward the concentration of dissolved O_2_ [35,37,39], while the slopes of the five K-L curves of T2-GaSbS differ somewhat (Figure 7d).

The transfer electron number (*n*) calculated from the slopes of corresponding linear K-L plots for NCF-3-Mn is around 3.95, while for T2-GaSbS, the electron transfer number ranges from 1.96 to 2.87, and the average number is 2.51, revealing that the NCF-3-Mn approaches the most efficient four-electron mechanism that involves the direct reduction of O_2_ to OH^−^ to avoid producing corrosive peroxide species during the ORR process, whereas the two-electron pathway that consists of reducing the O_2_ to HO^2−^ and further reduced to OH^−^ is dominative for T2-GaSbS towards the ORR [22,25,38].

On the whole, as summarized in Table S1, by comparing the ORR performance of NCF-3-Mn with other materials, it is notable that the NCF-3-Mn exhibits an outstanding ORR activity, ranking among the most active MCCF for ORRs to date in terms of *E*_onset_, *E*_1/2_, *j*_L_ and *n* and comparable to those of other recently reported Mn-based materials [14,15,16,17,29,30,31,32,36,37,38,40].

In view of the aforementioned experimental results, the good ORR activity of NCF-3-Mn is postulated to originate from the following two possible reasons: (1) The presence of Mn-N-C structures in the framework of NCF-3-Mn could serve as the exposed active sites for activating O_2_, optimizing the chemisorption of intermediates and subsequently breaking the O–O bond via reducing the energy barrier and thereby facilitating the electron transfer involved in ORRs, resembling the reported single transition metal atom or site-based M-N-C ORR electrocatalysts [14,15,16,17,18,22,23,25]. (2) The distinctive Mn-S bonds that linked [Mn(TEPA)]^2+^ complexes with supertetrahedral T3 clusters in NCF-3-Mn could serve as the highspeed channels for the oriented transmission of electrons from electron-rich aggregates—supertetrahedral T3 clusters to the [Mn(TEPA)]^2+^ complexes that consist of Mn-N-C structures, facilitating the ORR process, which resembles the polyoxometalate–metalloporphyrin organic framework for the highly selective electroreduction of CO_2_ [18,41,42].

## 4. Conclusions

In summary, we reported an MCCF (NCF-3-Mn) constructed from the single Sb^3+^ ion linked supertetrahedral T3 cluster of [Ga_10_SbS_19_SH] and TM complexes of [Mn(TEPA)]^2+^ and its application in an ORR for the first time. Significantly, compared with T2-GaSbS, the introduction of [Mn(TEPA)]^2+^ in NCF-3-Mn brings many Mn-S bonds and Mn-N-C structures, which could be the exposed active sites for activating O_2_ and subsequently breaking the O–O bond with a lower energy barrier and thereby facilitating the ORR. Thus, the as-synthesized NCF-3-Mn displayed efficient electrocatalytic performance with high onset and half-wave potentials (0.9 V vs. RHE), a relative small Tafel slope (73.5 mV/dec), quasi-four-electron transfer (*n* = 3.95) as well as good stability in 0.1 M KOH, which could be attributed to the effective electron transfer between the Mn-N-C structure and the supertetrahedral T3 cluster through the Mn-S bonds in the NCF-3-Mn framework. As the TM complexes and supertetrahedral clusters are multifarious, this work will inspire more related researchers to design and construct more novel MCCFs possessing potential excellent ORR activity as Pt-free ORR electrocatalysts for renewable energy conversion and storage [43] and to explore the in-depth structure–function relationships of these materials.

## Data Availability

Dataset available from the corresponding author upon request.

## Supplementary Materials

The following supporting information can be downloaded at: https://www.mdpi.com/article/10.3390/nano15151186/s1, Figure S1: Full-scan XPS spectrum of NCF-3-Mn; Figure S2: CV curves of (a) NCF-3-Mn and (b) T2-GaSbS at different scan rates in the potential window of 0.9813–1.0813 V vs. RHE.; Figure S3: Linear fitting of current density versus scan rate for NCF-3-Mn and T2-GaSbS; Figure S4: (a) LSV curves of T2-GaSbS before and after 1000 CV cycles over the potential range from 0.6 to 1.0 V vs. RHE; (b) Time-dependent current density curves of NCF-3-Mn under a static potential; Figure S5: Chronoamperometric response of NCF-3-Mn at 0.5 V vs. RHE after addition of 3.0 M methanol in O_2_-saturated 0.1 M KOH solution; Table S1: Comparison of the ORR performance of NCF-3-Mn with other MCCFs and recently reported Mn-based materials. Refs. [14,15,16,17,29,30,31,32,36,37,38,40,44,45,46,47] are cited in the Supplementary Materials File.

## References

[1] Li S., Shi L., Guo Y., Wang J., Liu D., Zhao S. (2024). Selective oxygen reduction reaction: Mechanism understanding, catalyst design and practical application. Chem. Sci..

[2] Zhai Q., Huang H., Lawson T., Xia Z., Giusto P., Antonietti M., Jaroniec M., Chhowalla M., Baek J.-B., Liu Y. (2024). Recent Advances on Carbon-Based Metal-Free Electrocatalysts for Energy and Chemical Conversions. Adv. Mater..

[3] Kment Š., Bakandritsos A., Tantis I., Kmentová H., Zuo Y., Henrotte O., Naldoni A., Otyepka M., Varma R.S., Zbořil R. (2024). Single Atom Catalysts Based on Earth-Abundant Metals for Energy-Related Applications. Chem. Rev..

[4] Du N., Roy C., Peach R., Turnbull M., Thiele S., Bock C. (2022). Anion-Exchange Membrane Water Electrolyzers. Chem. Rev..

[5] Yang Y., Peltier C.R., Zeng R., Schimmenti R., Li Q., Huang X., Yan Z., Potsi G., Selhorst R., Lu X. (2022). Electrocatalysis in Alkaline Media and Alkaline Membrane-Based Energy Technologies. Chem. Rev..

[6] Zhao Y., Adiyeri Saseendran D.P., Huang C., Triana C.A., Marks W.R., Chen H., Zhao H., Patzke G.R. (2023). Oxygen Evolution/Reduction Reaction Catalysts: From In Situ Monitoring and Reaction Mechanisms to Rational Design. Chem. Rev..

[7] Zhu Z., Jiang T., Ali M., Meng Y., Jin Y., Cui Y., Chen W. (2022). Rechargeable Batteries for Grid Scale Energy Storage. Chem. Rev..

[8] Shiva Kumar S., Lim H. (2022). An overview of water electrolysis technologies for green hydrogen production. Energy Rep..

[9] Liu Q., Wang L., Fu H. (2023). Research progress on the construction of synergistic electrocatalytic ORR/OER self-supporting cathodes for zinc–air batteries. J. Mater. Chem. A.

[10] Li J. (2022). Oxygen Evolution Reaction in Energy Conversion and Storage: Design Strategies Under and Beyond the Energy Scaling Relationship. Nano-Micro Lett..

[11] Kumar Y., Mooste M., Tammeveski K. (2023). Recent progress of transition metal-based bifunctional electrocatalysts for rechargeable zinc–air battery application. Curr. Opin. Electrochem..

[12] Sarapuu A., Lilloja J., Akula S., Zagal J.H., Specchia S., Tammeveski K. (2023). Recent Advances in Non-Precious Metal Single-Atom Electrocatalysts for Oxygen Reduction Reaction in Low-Temperature Polymer-Electrolyte Fuel Cells. ChemCatChem.

[13] Li X., Lei H., Xie L., Wang N., Zhang W., Cao R. (2022). Metalloporphyrins as Catalytic Models for Studying Hydrogen and Oxygen Evolution and Oxygen Reduction Reactions. Acc. Chem. Res..

[14] Luo Z., Xie J., Cheng J., Wei F., Lyu S., Zhu J., Shi X., Yang X., Wu B., Xu Z.J. (2025). Spin-State Manipulation of Atomic Manganese Center by Phosphide-Support Interactions for Enhanced Oxygen Reduction. Adv. Mater..

[15] Chen G., Qiu X., Liu S., Cui Y., Sun Y., Zhang Y., Liu Y., Liu G., Kim Y., Xing W. (2025). Mn–N–C with High-Density Atomically Dispersed Mn Active Sites for the Oxygen Reduction Reaction. Angew. Chem. Int. Ed..

[16] Zhong G., Zou L., Chi X., Meng Z., Chen Z., Li T., Huang Y., Fu X., Liao W., Zheng S. (2024). Atomically dispersed Mn–Nx catalysts derived from Mn-hexamine coordination frameworks for oxygen reduction reaction. Carbon Energy.

[17] Zhang L., Dong Y., Li L., Shi Y., Zhang Y., Wei L., Dong C.-L., Lin Z., Su J. (2024). Concurrently Boosting Activity and Stability of Oxygen Reduction Reaction Catalysts via Judiciously Crafting Fe–Mn Dual Atoms for Fuel Cells. Nano-Micro Lett..

[18] Pei J., Shang H., Mao J., Chen Z., Sui R., Zhang X., Zhou D., Wang Y., Zhang F., Zhu W. (2024). A replacement strategy for regulating local environment of single-atom Co-S_x_N_4−x_ catalysts to facilitate CO_2_ electroreduction. Nat. Commun..

[19] Lu X., Yang P., Wan Y., Zhang H., Xu H., Xiao L., Li R., Li Y., Zhang J., An M. (2023). Active site engineering toward atomically dispersed M−N−C catalysts for oxygen reduction reaction. Coord. Chem. Rev..

[20] Dong F., Wu M., Chen Z., Liu X., Zhang G., Qiao J., Sun S. (2021). Atomically Dispersed Transition Metal-Nitrogen-Carbon Bifunctional Oxygen Electrocatalysts for Zinc-Air Batteries: Recent Advances and Future Perspectives. Nano-Micro Lett..

[21] Gao C., Mu S., Yan R., Chen F., Ma T., Cao S., Li S., Ma L., Wang Y., Cheng C. (2022). Recent Advances in ZIF-Derived Atomic Metal–N–C Electrocatalysts for Oxygen Reduction Reaction: Synthetic Strategies, Active Centers, and Stabilities. Small.

[22] Yu S., Levell Z., Jiang Z., Zhao X., Liu Y. (2023). What Is the Rate-Limiting Step of Oxygen Reduction Reaction on Fe–N–C Catalysts?. J. Am. Chem. Soc..

[23] Zeng Y., Li C., Li B., Liang J., Zachman M.J., Cullen D.A., Hermann R.P., Alp E.E., Lavina B., Karakalos S. (2023). Tuning the thermal activation atmosphere breaks the activity–stability trade-off of Fe–N–C oxygen reduction fuel cell catalysts. Nat. Catal..

[24] Charalampopoulos G., Daletou M.K. (2025). Comparative development and evaluation of Fe–N–C electrocatalysts for the oxygen reduction reaction: The effect of pyrolysis and iron-bipyridine structures. Mater. Rep. Energy.

[25] Zhao X., Liu Y. (2021). Origin of Selective Production of Hydrogen Peroxide by Electrochemical Oxygen Reduction. J. Am. Chem. Soc..

[26] Zhang J., Feng P., Bu X., Wu T. (2021). Atomically precise metal chalcogenide supertetrahedral clusters: Frameworks to molecules, and structure to function. Natl. Sci. Rev..

[27] Zhang J., Bu X., Feng P., Wu T. (2020). Metal Chalcogenide Supertetrahedral Clusters: Synthetic Control over Assembly, Dispersibility, and Their Functional Applications. Acc. Chem. Res..

[28] Wang Z., Liu J.-X., Ma H., Xu Y.-L., Zhou R., Li D.-S., Yuan S.-F., Wu T. (2024). Atomic- and molecular-level modulation of Mn^2+^-related emission using atomically-precise metal chalcogenide semiconductor nanoclusters. Coord. Chem. Rev..

[29] Sun L., Wang X.-X., Su F. (2023). Synthesis, structure, and electrocatalytic oxygen reduction reaction properties of metal chalcogenide non-supertetrahedral In-Sn-S cluster materials. Chin. J. Inorg. Chem.

[30] Wang X.X., Guo Y.N., Su F., Han C., Sun L. (2024). Synthesis, structure, and electrocatalytic oxygen reduction reaction properties of metal antimony based chalcogenide clusters. Chin. J. Inorg. Chem.

[31] Zhang Y., Hu D., Xue C., Yang H., Wang X., Wu T. (2018). A 3D neutral chalcogenide framework built from a supertetrahedral T3 cluster and a metal complex for the electrocatalytic oxygen reduction reaction. Dalton Trans..

[32] Zhang X., Wang S., Ding Z., Zhang H., Ma X., Zhou X., Ma Y., Mu Y., Yu J., Huang T. (2025). The structure and catalytic performance for oxygen reduction reaction of worm-like CNTs with Fe_3_P and Fe_0.8_Mn_0.2_ alloy encapsulated. J. Electroanal. Chem..

[33] Li J., Liu C., Wang X., Ding Y., Wu Z., Sun P., Tang J., Zhang J., Li D.-S., Chen N. (2022). Stable 3D neutral gallium thioantimonate frameworks decorated with transition metal complexes for a tunable photocatalytic hydrogen evolution. Dalton Trans..

[34] Zhang B., Li W.-A., Liao Y.-Y., Zhang C., Feng M.-L., Huang X.-Y. (2018). [CH_3_NH_3_]_4_Ga_4_SbS_9_S_0.28_O_0.72_H: A Three-Dimensionally Open-Framework Heterometallic Chalcogenidoantimonate Exhibiting Ni^2+^ Ion-Exchange Property. Chem-Asian J..

[35] Zhang Y., Wang X., Hu D., Xue C., Wang W., Yang H., Li D., Wu T. (2018). Monodisperse Ultrasmall Manganese-Doped Multimetallic Oxysulfide Nanoparticles as Highly Efficient Oxygen Reduction Electrocatalyst. ACS Appl. Mater. Interfaces.

[36] Sun P., Wu J., Wang Z., Wang X., Chen N., Wu T. (2021). A pillar-layered chalcogenide framework assembled by [Mn_5_S_12_N_12_]_n_ layers and [Sb_2_S_5_] inorganic pillars. Dalton Trans..

[37] Wang X.-L., Wu Z., Wang X., Xue C., Liu C., Zhang J., Zhou R., Li D.-S., Wu T. (2021). Bifunctional electrocatalysts derived from cluster-based ternary sulfides for oxygen electrode reactions. Electrochim. Acta.

[38] Flores-Lasluisa J.X., Carré B., Caucheteux J., Compère P., Léonard A.F., Job N. (2024). Development of In Situ Methods for Preparing La-Mn-Co-Based Compounds over Carbon Xerogel for Oxygen Reduction Reaction in an Alkaline Medium. Nanomaterials.

[39] Pan H., Wang X.-L., Li F., Xu Q. (2023). A one-stone-two-birds strategy to construct metal–organic framework-derived cobalt phosphide as an efficient bifunctional electrocatalyst for oxygen electrode reactions. J. Mater. Chem. A.

[40] Lin J., Dong Y., Zhang Q., Hu D., Li N., Wang L., Liu Y., Wu T. (2015). Interrupted Chalcogenide-Based Zeolite-Analogue Semiconductor: Atomically Precise Doping for Tunable Electro-/Photoelectrochemical Properties. Angew. Chem. Int. Ed..

[41] Huang Q., Liu J., Feng L., Wang Q., Guan W., Dong L.-Z., Zhang L., Yan L.-K., Lan Y.-Q., Zhou H.-C. (2019). Multielectron transportation of polyoxometalate-grafted metalloporphyrin coordination frameworks for selective CO_2_-to-CH_4_ photoconversion. Natl. Sci. Rev..

[42] Wang Y.-R., Huang Q., He C.-T., Chen Y., Liu J., Shen F.-C., Lan Y.-Q. (2018). Oriented electron transmission in polyoxometalate-metalloporphyrin organic framework for highly selective electroreduction of CO_2_. Nat. Commun..

[43] Zhu Y., Su C., Xu X., Zhou W., Ran R., Shao Z. (2014). A Universal and Facile Way for the Development of Superior Bifunctional Electrocatalysts for Oxygen Reduction and Evolution Reactions Utilizing the Synergistic Effect. Chem. Eur. J..

[44] Wu Z., Wang X.-L., Hu D., Wu S., Liu C., Wang X., Zhou R., Li D.-S., Wu T. (2019). A new cluster-based chalcogenide zeolite analogue with a large inter-cluster bridging angle. Inorg. Chem. Front..

[45] Wang W., Wang X., Zhang J., Yang H., Luo M., Xue C., Lin Z., Wu T. (2019). Three-Dimensional Superlattices Based on Unusual Chalcogenide Supertetrahedral In–Sn–S Nanoclusters. Inorg. Chem..

[46] Wang W., Wang X., Hu D., Yang H., Xue C., Lin Z., Wu T. (2018). An Unusual Metal Chalcogenide Zeolitic Framework Built from the Extended Spiro-5 Units with Supertetrahedral Clusters as Nodes. Inorg. Chem..

[47] Lv J., Zhang J., Xue C., Hu D., Wang X., Li D.-S., Wu T. (2019). Two Penta-Supertetrahedral Cluster-Based Chalcogenide Open Frameworks: Effect of the Cluster Spatial Connectivity on the Electron-Transport Efficiency. Inorg. Chem..

