# Supplementary material for: A Stable Metal Chalcogenide Cluster-Based Framework Decorated with Transition Metal Complexes for an Efficient Electrocatalytic O2 Reduction Reaction
Source: Nanomaterials (Basel). 2025 Aug 1;15(15):1186. doi: 10.3390/nano15151186 (PMC12348372; doi:10.3390/nano15151186)
Supplement: Supplementary file 1 [file nanomaterials-15-01186-s001.zip › nanomaterials-3780839-supplementary.pdf]

Supporting Information for

A Stable Metal Chalcogenide Cluster-based Framework Decorated with Transition Metal Complexes for Efficient Electrocatalytic O_2 Reduction Reaction

Xiang Wang [1,2,†,*], Juan Li [4,†], and Tao Wu [3,*]

[1] College of New Energy Materials and Chemistry, Leshan Normal University, Leshan 614000, China.

[2] Leshan West Silicon Materials Photovoltaic and New Energy Industry Technology Research Institute, Leshan 614000, China.

[3] College of Chemistry and Materials Science, and Guangdong Provincial Key Laboratory of Functional Supramolecular Coordination Materials and Applications, Jinan University, Guangzhou 510632, China.

[4] Hongguang Middle School, Nanjing Jiangsu 210000, China.

* Corresponding author E-mail: *3810552437@qq.com* (X.W.)*; wutao@jnu.edu.cn* (T.W.)

† These authors contributed equally to this work.

Experimental

Electrochemical measurements

All the electrochemical measurements are conducted on a rotating ring disk electrode (RRDE) apparatus (RRDE-3A, BAS Inc.) For ORR, cyclic voltammetry (CV) measurements were carried out in N_2/O_2 saturated 0.1 M KOH solution with a scan rate of 50 mV·s^{-1}, Linear sweep voltammetry (LSV) tests were carried out in O_2 saturated 0.1 M KOH solution with a sweep rate of 5 mV·s^{-1} under different rotation speeds varying from 625 to 2500 rpm, chronoamperometry (CA) tests were performed at 0.5 V (*vs.* RHE) in O_2-saturated 0.1 M KOH solution, electrochemically active surface area (ECSA) was calculated by measuring the double layer capacitance (C_{dl}) under the potential window of 0.981-1.081 V (*vs.* RHE) with various scan rates (5-30 mV·s^{-1}), the long-term stability was measured by the LSV curve after an accelerated durability test (ADT) over the potential range from 0.6 to 1.0 V (*vs.* RHE) with a scan rate of 50 mV·s^{-1} in O_2-saturated 0.1 M KOH solution for 1000 cycles. In addition, the methanol tolerance test was performed via a CA test with the addition of 3.0 M methanol. The electron transfer number (*n*) during the ORR was determined at various potentials according to the Koutecky-Levich (K-L) Equation (S1)-(S3):

$$\frac{1}{j} = \frac{1}{j_L} + \frac{1}{j_K} = \frac{1}{B\omega^{1/2}} + \frac{1}{j_K} \quad \text{(S1)}$$

$$B = 0.62nFC_0D_0^{2/3}v^{-1/6} \quad \text{(S2)}$$

$$j_K = nFkC_0 \quad \text{(S3)}$$

where j is the measured current density, j_K and j_L are the kinetic and diffusion-limiting current densities. B is the reciprocal of the slope of K-L plots. ω is the rotation rate (ω=2πN, N is the linear rotation speed), F is the Faraday constant (F=96485 C·mol^{-1}), C_0 is the bulk concentration of O_2 (1.2×10^{-6} mol·cm^{-3}), D_0 is the diffusion coefficient of O_2 in 0.1 M KOH (1.9×10^{-5} cm^2·s^{-1}), ν is the kinetic viscosity of the electrolyte (0.01 cm^2·s^{-1}), and k is the electron-transfer rate constant.

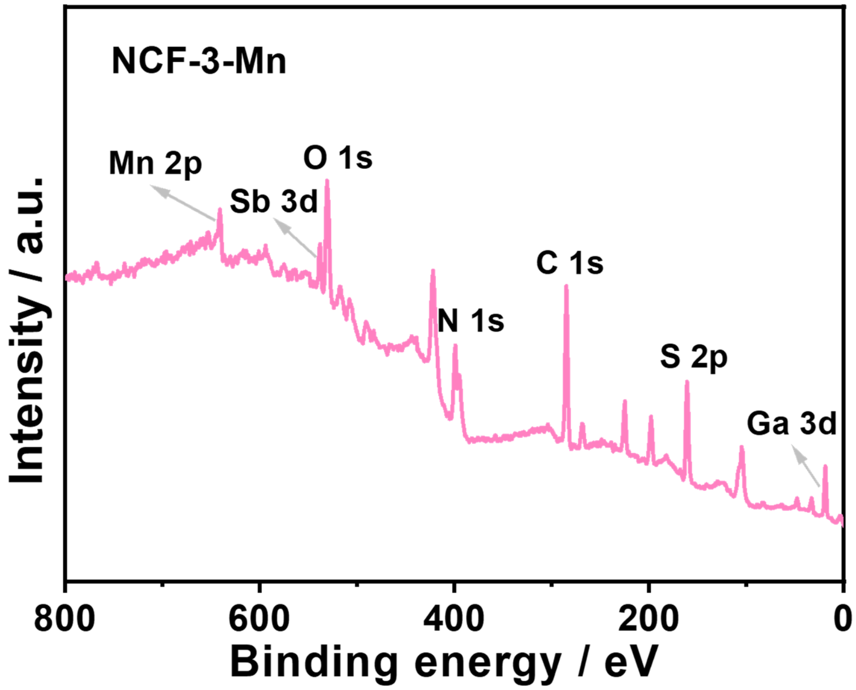

Figure S1. Full-scan XPS spectrum of NCF-3-Mn.

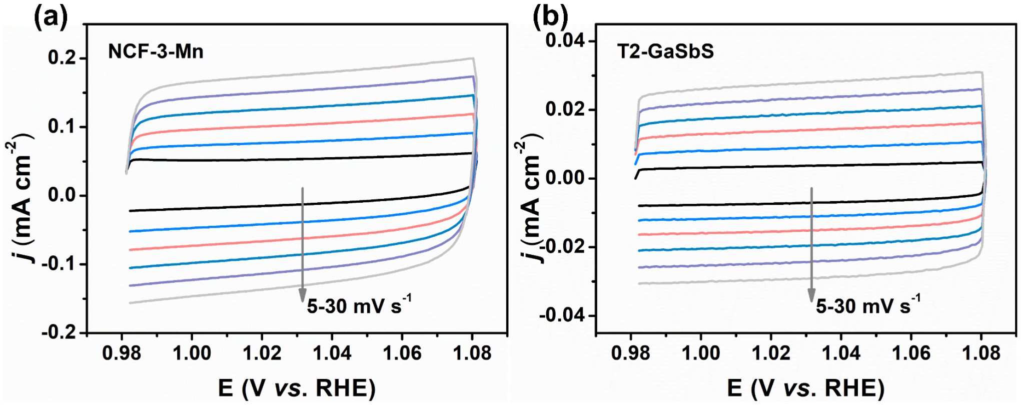

Figure S2. CV curves of (a) NCF-3-Mn and (b) T2-GaSbS at different scan rates in the potential window of 0.9813-1.0813 V *vs*. RHE.

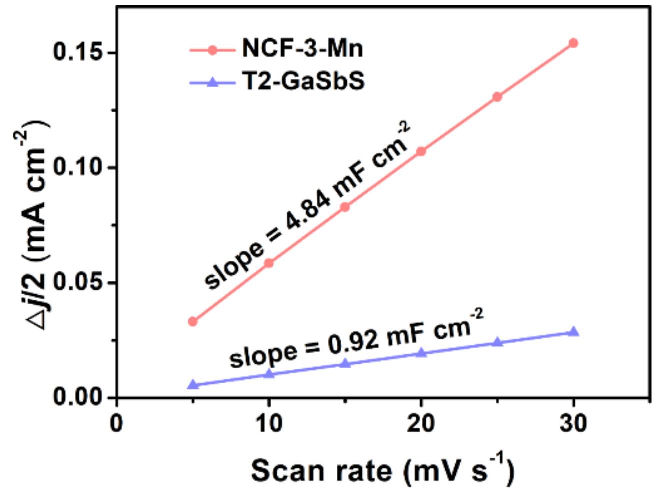

Figure S3. Linear fitting of current density versus scan rate for NCF-3-Mn and T2-GaSbS.

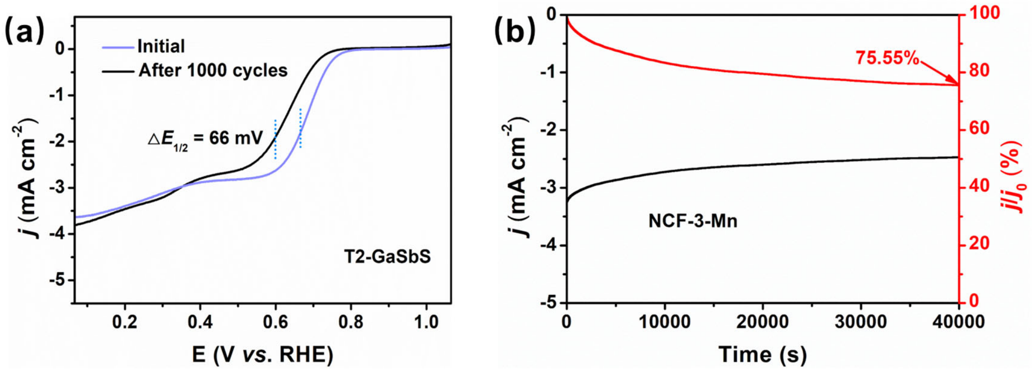

Figure S4. (a) LSV curves of T2-GaSbS before and after 1000 CV cycles over the potential range from 0.6 to 1.0 V vs. RHE; (b) Time-dependent current density curves of NCF-3-Mn under a static potential.

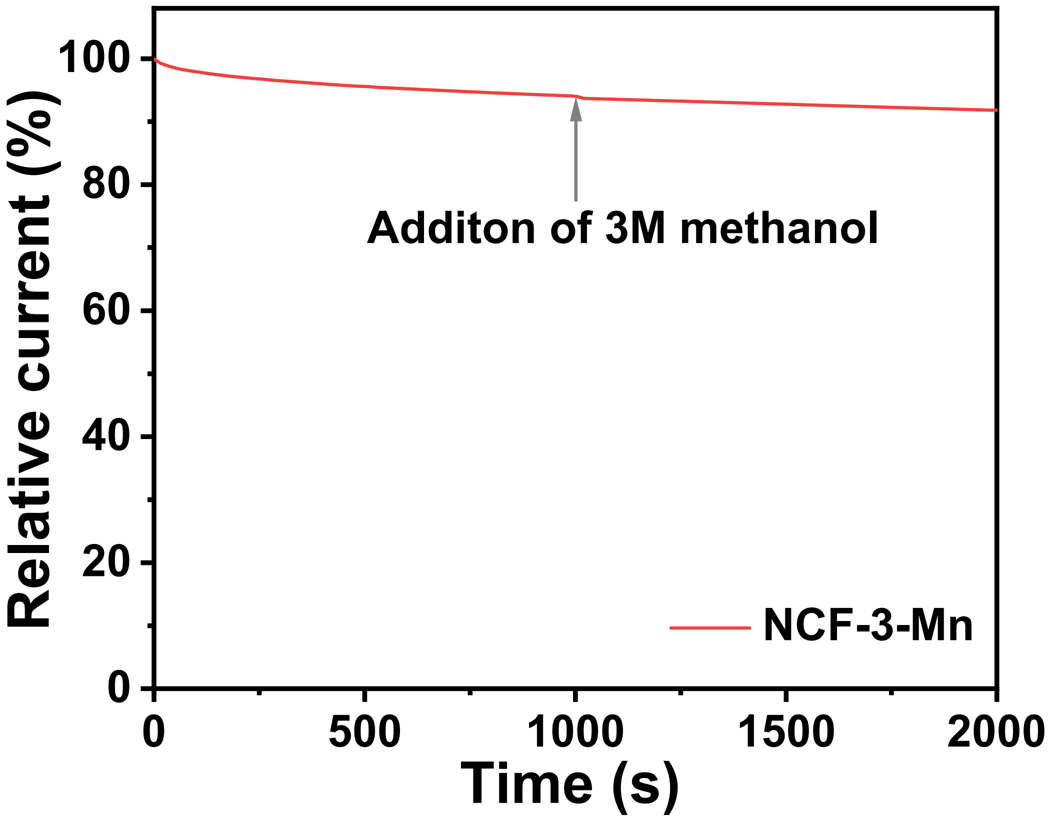

Figure S5. Chronoamperometric response of NCF-3-Mn at 0.5 V *vs.* RHE after addition of 3.0 M methanol in O_2-saturated 0.1 M KOH solution.

Table S1. Comparison the ORR performance of NCF-3-Mn with other MCCFs and recently reported Mn-based materials.

**Catalysts**	E_{onset} (V *vs*. RHE) (j = -0.1 mA·cm^{-2})	$E_{1/2}$ (V *vs*. RHE)	**Electron-transfer number (*n*)**	**Diffusion-limited current density (|j_L|) (mA/cm^2)**	**References**
**NCF-3-Mn**	**0.90**	**0.73**	**3.95**	**3.98**	**This work**
NCF-4	0.87	0.76	3.74	3.8	S1[1]
$(C_5H_{12}N)_2[In_2Sb_2S_7]$	0.73	0.62	3.6	4.21	S2[2]
$[Sb_4S_5(S_3)]·C_5H_{11}N$	0.68	0.60	1.9	2.24	
$[Mn_5Sb_6S_{15}(N_2H_4)_6]·(H_2en)·DMF·0.5N_2H_4$	0.89	0.73	3.3	4.5	S3[3]
$(C_7H_{13}N_2)[InS_2]$	0.69	0.66	1.7	2.1	S4[4]
$(C_7H_{13}N_2)_4[In_2S_{11}Sn_3]$	0.72	0.69	2.4	2.7	
$(C_7H_{13}N_2)_3[In_3S_{12}Sn_3]$	0.77	0.67	3.6	4	
SOF-20	0.82	0.72	2.8	2.7	S5[5]
SOF-21	0.83	0.72	2.6	2.8	
SOF-25	0.76	0.67	2.2	2.9	S6[6]
SOF-28	0.77	0.68	2.6	3.6	
SOF-27	0.79	0.7	2.8	2.8	S7[7]
MCOF-1	0.77	0.7	3.36	2.7	S8[8]
MOCF-2	0.85	0.68	3.51	4.4	
CSZ-5-InSe	0.76	0.68	2.2	2.8	S9[9]
MnInS/NH_2-CNT	0.82	0.72	-	2.9	S10[10]
P_CX_5.6	0.85	0.72	3.68	4.58	S11[11]
(FeMn-DA)–N–C	1.02	0.92	3.95	5.9	S12[12]
MoP@Mn_{SAC}-NC	1.01	0.894	4	5.9	S13[13]
FP/FM@CNTs	0.97	0.892	3.3	4	S14[14]
Mn-N-C-4.5	0.92	0.89	4	5	S15[15]
MnNCS-4-800	0.99	0.89	3.7	5	S16[16]

-: not mentioned.

Supplementary References

1. Zhang, Y.; Hu, D.; Xue, C.; Yang, H.; Wang, X.; Wu, T., A 3D neutral chalcogenide framework built from a supertetrahedral T3 cluster and a metal complex for the electrocatalytic oxygen reduction reaction. *Dalton Trans.* **2018**, *47*, 3227-3230.
2. Sun, L.; Wang, X.-X.; Su, F., Synthesis, structure, and electrocatalytic oxygen reduction reaction properties of metal chalcogenide non-supertetrahedral In-Sn-S cluster materials. *Chin. J. Inorg. Chem* **2023**, *39*, 1369-1378.
3. Sun, P.; Wu, J.; Wang, Z.; Wang, X.; Chen, N.; Wu, T., A pillar-layered chalcogenide framework assembled by [$Mn_5S_{12}N_{12}$]n layers and [Sb_2S_5] inorganic pillars. *Dalton Trans.* **2021**, *50*, 16473-16477.
4. Wang, X. X.; Guo, Y. N.; Su, F.; Han, C.; Sun, L., Synthesis, structure, and electrocatalytic oxygen reduction reaction properties of metal antimony.based chalcogenide clusters. *Chin. J. Inorg. Chem* **2024**, *40*, 1201-1208.
5. Wu, Z.; Wang, X.-L.; Hu, D.; Wu, S.; Liu, C.; Wang, X.; Zhou, R.; Li, D.-S.; Wu, T., A new cluster-based chalcogenide zeolite analogue with a large inter-cluster bridging angle. *Inorg. Chem. Front.* **2019**, *6*, 3063-3069.
6. Wang, W.; Wang, X.; Zhang, J.; Yang, H.; Luo, M.; Xue, C.; Lin, Z.; Wu, T., Three-Dimensional Superlattices Based on Unusual Chalcogenide Supertetrahedral In–Sn–S Nanoclusters. *Inorg. Chem.* **2019**, *58*, 31-34.
7. Wang, W.; Wang, X.; Hu, D.; Yang, H.; Xue, C.; Lin, Z.; Wu, T., An Unusual Metal Chalcogenide Zeolitic Framework Built from the Extended Spiro-5 Units with Supertetrahedral Clusters as Nodes. *Inorg. Chem.* **2018**, *57*, 921-925.
8. Lv, J.; Zhang, J.; Xue, C.; Hu, D.; Wang, X.; Li, D.-S.; Wu, T., Two Penta-Supertetrahedral Cluster-Based Chalcogenide Open Frameworks: Effect of the Cluster Spatial Connectivity on the Electron-Transport Efficiency. *Inorg. Chem.* **2019**, *58*, 3582-3585.
9. Lin, J.; Dong, Y.; Zhang, Q.; Hu, D.; Li, N.; Wang, L.; Liu, Y.; Wu, T., Interrupted Chalcogenide-Based Zeolite-Analogue Semiconductor: Atomically Precise Doping for Tunable Electro-/Photoelectrochemical Properties. *Angew. Chem. Int. Ed.* **2015**, *54*, 5103-5107.
10. Wang, X.-L.; Wu, Z.; Wang, X.; Xue, C.; Liu, C.; Zhang, J.; Zhou, R.; Li, D.-S.; Wu, T., Bifunctional electrocatalysts derived from cluster-based ternary sulfides for oxygen electrode reactions. *Electrochim. Acta* **2021**, *376*, 138048.
11. Flores-Lasluisa, J. X.; Carré, B.; Caucheteux, J.; Compère, P.; Léonard, A. F.; Job, N., Development of In Situ Methods for Preparing La-Mn-Co-Based Compounds over Carbon Xerogel for Oxygen Reduction Reaction in an Alkaline Medium. *Nanomaterials* **2024**, *14*, 1362.
12. Zhang, L.; Dong, Y.; Li, L.; Shi, Y.; Zhang, Y.; Wei, L.; Dong, C.-L.; Lin, Z.; Su, J., Concurrently Boosting Activity and Stability of Oxygen Reduction Reaction Catalysts via Judiciously Crafting Fe–Mn Dual Atoms for Fuel Cells. *Nano-Micro Lett.* **2024**, *17*, 88.
13. Luo, Z.; Xie, J.; Cheng, J.; Wei, F.; Lyu, S.; Zhu, J.; Shi, X.; Yang, X.; Wu, B.; Xu, Z. J., Spin-State Manipulation of Atomic Manganese Center by Phosphide-Support Interactions for Enhanced Oxygen Reduction. *Adv. Mater.* **2025**, 2504585.
14. Zhang, X.; Wang, S.; Ding, Z.; Zhang, H.; Ma, X.; Zhou, X.; Ma, Y.; Mu, Y.; Yu, J.; Huang, T., The structure and catalytic performance for oxygen reduction reaction of worm-like CNTs with

Fe_3P and $Fe_{0.8}Mn_{0.2}$ alloy encapsulated. *J. Electroanal. Chem.* **2025**, *990*, 119175.
15. Chen, G.; Qiu, X.; Liu, S.; Cui, Y.; Sun, Y.; Zhang, Y.; Liu, Y.; Liu, G.; Kim, Y.; Xing, W.; Wang, H.; Shao, M., Mn–N–C with High-Density Atomically Dispersed Mn Active Sites for the Oxygen Reduction Reaction. *Angew. Chem. Int. Ed.* **2025**, *64*, e202503934.
16. Zhong, G.; Zou, L.; Chi, X.; Meng, Z.; Chen, Z.; Li, T.; Huang, Y.; Fu, X.; Liao, W.; Zheng, S.; Xu, Y.; Peng, F.; Peng, X., Atomically dispersed Mn–N_x catalysts derived from Mn-hexamine coordination frameworks for oxygen reduction reaction. *Carbon Energy* **2024**, *6*, e484.
